# Iron deficiency anemia and thrombosis risk in children—revisiting an old hypothesis

**DOI:** 10.3389/fped.2022.926925

**Published:** 2022-08-01

**Authors:** Hannah Kalff, Holger Cario, Susanne Holzhauer

**Affiliations:** ^1^Department of Pediatric Hematology and Oncology, Charité University Medicine, Berlin, Germany; ^2^Department of Pediatrics and Adolescent Medicine, University Medical Center Ulm, Ulm, Germany

**Keywords:** anemia, pediatric thrombosis, iron deficiency, cerebral sinus venous thrombosis (CVST), iron deficiency anemia (IDA)

## Abstract

Iron deficiency anemia has a high prevalence in children and has repeatedly been implicated as a risk factor for arterial and venous thrombosis. As an effective therapy for iron deficiency anemia is available, understanding the association between this form of anemia and the potentially severe thrombosis phenotype is of major clinical interest. Recent findings shed light on pathophysiology of hypercoagulability resulting from iron-restricted erythropoiesis. Specifically, an animal model of induced iron deficiency allowed identifying multiple mechanisms, by which iron deficiency anemia results in increased thrombus formation and thrombus progression both in arterial and venous thrombosis. These findings complement and support conclusions derived from clinical data. The purpose of this mini review is to summarize current evidence on the association of iron deficiency anemia and thrombosis. We want to increase the awareness of iron deficiency as a risk factor for thrombosis in the pediatric population. We discuss how novel pathophysiological concepts can be translated into the clinical settings and suggest clinical studies on prevention and treatment strategies in high-risk patient groups.

## Introduction

Iron deficiency (ID) is the most common micronutrient deficiency worldwide and one main cause of anemia, affecting one third of the world population (1–4). Iron deficiency anemia (IDA) affects ~40% of children under 5 years of age (5). IDA usually manifests from late infancy onwards, when iron reserves built up during the fetal period are depleted and increased requirements arise during rapid growth. A second peak occurs in girls during puberty, when the growth spurt meets the blood losses associated with menstruation. Poor diet or increased consumption of cow milk contribute to IDA in pediatric patients. Because of the low iron content of cow milk, supplementation is often necessary to meet the body's iron requirements (6). Additionally, cow milk can result in occult stool blood loss, by inducing clinically silent enterocolitis (7).

Anemia, and specifically IDA, has long been implicated as a risk factor for arterial and venous thrombosis. This observation was initially made in individual patients or small case-series. IDA is not established as a prothrombotic risk factor in clinical practice. A lack of randomized studies on this question and a shortage of experimental data on the pathophysiological mechanisms aggravate this problem.

A recently established rodent model of iron deficiency advanced our understanding of pathophysiological mechanisms of thrombus formation and propagation in iron deficiency (8). Identifying relevant biological factors in this association is crucial for appropriate management and preventive strategies. Jimenez et al. (8) showed an association between IDA, reactive thrombocytosis and increased thrombotic tendency. The results establish IDA as an independent risk factor for thrombosis and show that thrombocytosis further ads to that risk in that model system.

Given the high prevalence of IDA in children, we need to better understand to what extent the association of IDA and thrombosis risk applies to children. The novel evidence should raise awareness among clinicians to identify high-risk groups that may benefit from therapeutic interventions.

## Pathophysiology

Using a rat model of dietary ID, K. Jimenez et al. recently showed that ID is an independent risk factor for both the development and propagation of thrombosis in rats (8). The researchers induced arterial and venous thrombosis in iron-deficient as well as control fed rats and measured thrombus size and structure histologically and via high frequency ultrasound. Thrombus formation, thrombus growth and final dimensions, the number of involved platelets as well as platelet activity were largest in iron-deficient rats. ID consistently induced thrombocytosis in this model. Additionally, the investigators found a higher baseline expression of the cell-adhesion molecule P-selectin by platelets in state of ID. These data suggest, that not only the number of circulating platelets, but also the level of activation of platelets is increased (8). Besides augmented clot formation, clot strength was also increased in iron-deficient rats. These results support the clinical observation, that IDA, independent of platelet numbers, is a risk factor for thrombosis. Additionally, these findings match with the results from a cross-sectional study using rotational thromboelastography that showed increased clot firmness as well as impaired thrombolysis in children with IDA (9). The rat model offers unique opportunities to explore in depth pathophysiological mechanisms resulting in the prothrombotic state observed in state of ID and the impact of thrombocytosis (10).

Thrombocytosis has repeatedly been implicated as the major driver of thrombosis in ID. Iron status has recently been reported as critical for regulating the lineage commitment of megakaryocytic/erythroid progenitors (MEP). Platelets originate from bipotent megakaryocytic/ erythroid progenitors. Using acquired (low iron diet) and genetic (Tmprss6/) mouse models of iron deficiency, Xavier-Ferrucio et al. demonstrated that lack of iron led to a shift toward megakaryocyte progenitors and subsequent thrombopoiesis at the expense of red cell production (11). During evolution, thrombocytosis may have conferred a selective hemostatic advantage in major bleeding, whereas reducing erythroid differentiation could preserve iron stores for essential functions in DNA synthesis, respiratory chain proteins and repair proteins. However, the underlying pathomechanisms are not completely understood, and we do not know, why only some but not all patients with IDA develop thrombocytosis.

**Figure 1:**
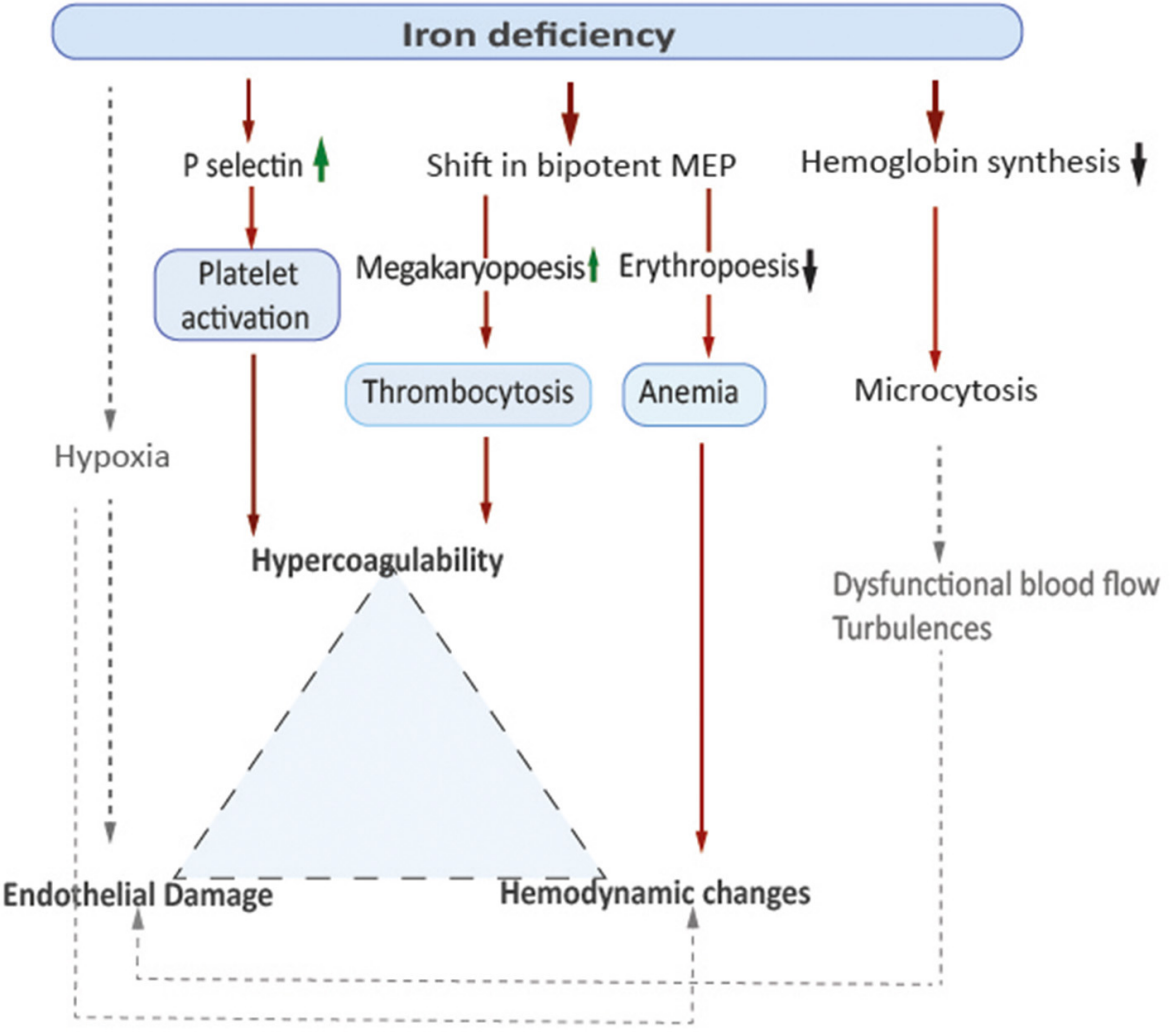
Model on pathophysiological concepts.

In addition to reactive secondary thrombocytosis and platelet activation demonstrated in the animal model, anemia itself might contribute to a prothrombotic state by affecting flow patterns within the vessels. Low hemoglobin-levels and decreased oxygen-carrying capacity of the blood lead to insufficient tissue supply, which is particularly relevant in situations of increased metabolic demands, such as infections. Increased blood flow velocity to compensate anemic hypoxia may result in hemodynamic changes such as turbulences that might result in endothelial damage and inflammation (12). In 2007, Aliefendioglu et al. measured cerebral blood flow velocities in 36 children with IDA using Doppler sonography and found a significant increase especially in in the observed group of 23 children with severe anemia defined as hemoglobin levels <10 g/dl (13). The researchers considered increased cardiac stroke volume as well as decreased vascular resistance as underlying reasons (13). Microcytic erythrocytes as seen in IDA are characterized by reduced cell-deformability associated with dysfunctional blood flow. This can increase blood viscosity and lead to activation of the coagulation cascade (14). Elevated factor VIII levels have been reported in patients with IDA associated thrombosis. In a case control study from Ozdemir et al. blood samples from 57 children with IDA and 48 healthy controls were compared, showing samples from children with IDA had shorter clot formation times and higher mean clot firmness. These data suggest higher tendency to coagulate, although results of thrombelastography remained in normal range (9). For a model on pathophysiological concepts, see Figure 1.

## Evidence from clinical studies

Although the association of IDA and thrombosis is well-recognized, data describing the clinical relevance, thrombosis location or specific patient-groups at risk are limited. So far, it has not been shown in clinical studies to which extent the elevated risk for thrombosis in iron deficiency can be attributed to iron deficiency, anemia, or secondary thrombocytosis. Moreover, studies included in this review used different definitions and cutoff values for ferritin or hemoglobin for evaluating the presence of ID and IDA (15).

Using large-volume clinical data from an institutional research database including 36,327 adults with IDA, Song et al. estimated a 2-fold increased thrombotic risk associated with reactive thrombocytosis in patients with IDA compared to patients with IDA and no thrombocytosis (16). Different to preceding studies the authors excluded patients in whom thrombocytosis was attributed to diagnoses other than ID. In children, IDA is a common condition, while both arterial and venous thrombosis are very rare in the general population. Thus, high quality population based or interventional studies are hardly feasible. Most data published on the association of IDA and thrombosis stem from case reports of individual patients or small case-series of children (14, 17–22). Most reports present arterial or venous thrombosis at unusual locations and in otherwise healthy children in whom IDA was an identified risk factor. These reports suggest that IDA contributes to thrombosis development in pediatric patients.

## Thrombus location

The association of IDA and thrombosis has been systematically addressed for arterial and venous thrombosis of the cerebral vessels. The evidence available for thrombosis located outside of the brain is scarce. In large cohorts of pediatric patients with venous thromboembolism, 9–13% were affected by cerebral sinovenous thrombosis (23, 24). Population based epidemiological data on incidence of specific thrombus locations in the state of ID and IDA are lacking, thus we can only speculate why cerebral thrombus location is reported most frequent as compared to other locations in children with ID and IDA. Thromboses of the extremities, otherwise the most common thrombosis location in children, predominantly occur in severely ill patients with central venous lines or arterial catheterization. In these children, evaluation of IDA may either be hampered by acute phase reactions disguising IDA or simply not be reported as another risk factor (25). A predominance of a specific thrombus locations has been described for other conditions and primarily in adults, e.g., for splanchnic vein thromboses in paroxysmal nocturnal hemoglobinuria (26), cerebral sinus venous thrombosis (CSVT) in pregnant women (27) or in patients with chronic hemolytic anemia (28).

## Cerebral thrombosis

The strongest evidence found on the association of IDA and thrombosis in children is provided by two case-control studies that examined the association of IDA and stroke in 36 children and 143 healthy controls (29, 30). In both studies, IDA was defined as serum ferritin levels below 12 μg/L with hemoglobin and mean corpuscular volume below reference ranges according to age and sex. Symptomatic children with arterial ischemic stroke or cerebral sinus venous thrombosis confirmed by computed tomography or MRI scan were included as cases in both studies. Thrombocytosis was defined as platelet counts > 450 × 10^9^/L. IDA was a significant risk factor for the development of stroke in both studies. In 2007, Maguire et al. indicated that previously healthy children with a stroke were twelve times more likely to be iron-deficient than controls. This association remained significant after adjusting for platelet counts with an odds ratio of 10 (CI: 3–33), indicating that the risk cannot be completely attributed to secondary thrombocytosis, although the latter was five times more common in cases that in controls (29). Azab et al. confirmed IDA as an independent risk factor with an odds ratio of 3.8 (CI: 1.3–11.2), again also in the patients included in this study, thrombocytosis was significantly more common in cases with IDA than in children with stroke without IDA (OR 10.5; CI: 1–152; *p* = 0.02). Relative risks differed between the two studies, most likely resulting from the small number of patients included in both studies and differences between study populations not reported (30). Importantly, CSVT predominated compared to arterial ischemic infarction in cases with IDA in both groups (29).

Further evidence stems from case series or case reports. A Swedish retrospective case-series from 2010 investigated risk factors of childhood stroke and found IDA and thrombocytosis in five of 51 children (31). Three of them were diagnosed with CSVT, the other two suffered from arterial ischemic stroke. Three other case-series examined risk factors for occurrence of CSVT in altogether 93 children from 0 to 18 years (32–34). They all named anemia, especially IDA, as a common condition or even predisposing risk factor for thrombosis of the cerebral veins. Numerous case-reports investigate the association of cerebral sinus venous thrombosis and iron deficiency anemia in otherwise healthy children. Specifically, excessive intake of milk or poor nutrition have been reported in these children (14, 20, 22, 35–40).

## Extra-cerebral venous and arterial thrombosis

Systematic data on the association of arterial or venous thromboses located in the extremities, abdomen or other locations and IDA in children are lacking. The available information stem from anecdotal case-reports presenting children with comorbidities that make it difficult to interpret the impact of anemia in these cases.

## Management and outcome

Standard of care to treat venous thrombosis or pulmonary embolism is therapeutic anticoagulation according to current treatment guidelines for VTE in neonates, children, and adolescents (41). In general, therapeutic anticoagulation is recommended for 3 months and extended therapy in case of a continuing increased thrombosis risk profile. Recently, Goldenberg et al. showed in the Kids-Dott trial, that for a selected subgroup of pediatric patients with provoked VTE, a shortened anticoagulant treatment for 6 weeks was not inferior (42). The definition of provoked was left to the treating physician in that trial, thus encouraging shortened treatment in children with ID/IDA associated thrombosis after correction of ID as well.

Additional, iron replacement therapy to correct for ID and anemia has regularly been used to restore iron in children with IDA and associated thrombosis. In adults, data suggests that anemia treatment results in both better outcome and reduction of recurrence. Supporting earlier studies, in a recent prospective study anemia was found to be an independent predictor of poor clinical outcome (adjusted OR 1.9, 95% CI 1.2–3.2) and mortality (adjusted OR 1.4, 95%) (43, 44). The influence of ID or IDA on outcome in children is debated. Sebire et al. (32) studied the impact of hemoglobin levels in a study on longterm outcome in 42 children with CSVT from five European pediatric neurology stroke registries. Hemoglobin levels were significantly higher at original presentation in those children with recanalization at follow-up than in those with partial response or persistent occlusion (*P* = 0.02), but anemia or microcytosis were no predictors of either death or neurological outcome.

To ensure the fastest and most reliable correction for ID and IDA in case of a thromboembolic event, we recommend intravenous iron substitution over the gradual correction of iron stores by oral iron preparations in these children (3). New intravenous iron formulations are available, that allow complete replacement dosing in one or two applications and with favorable safety profiles (2, 45). Whether patients benefit from erythrocyte transfusion therapy to correct for ID or anemia in case of an acute thrombotic event is uncertain (46). Transfusions have multiple potentially deleterious effects including platelet activation of the plasmatic coagulation or disruption of nitric oxide mediated vasodilatation. Moreover, red blood cell transfusions are associated with thrombosis development in adults (47).

In adults, ID but not anemia has been shown to be predictive for recurrent thrombosis in a prospective cohort study following 229 patients after a first thromboembolic event with a hazard ratio of 3.2 for ID (48). However, no evidence in children is available on ID/ IDA and recurrence of thrombosis. We need further investigations in large and well-characterized patient collectives with matched controls on the management of anemia in thrombosis. However, given the low incidence of thrombosis in children, clinical trials are difficult to perform.

## High-risk patient groups

Strategies for primary prevention of VTE in high-risk collectives come more into focus. Children and adolescents with inflammatory bowel disease (IBD), chronic kidney failure, cancer, or hematological malignancy carry an increased risk of developing thrombosis. These patient groups often have anemia as comorbidity, herewith potentially further increasing thrombosis risk.

In children with IBD, the risk of thrombosis was substantially increased compared with that of the general pediatric population (49), with relative risks ranging from 14 to 22 (49, 50). The proportion of CSVT was particularly high in these pediatric patients with IBD with 50% of all sites of venous thrombosis as calculated in a meta-analysis and recently confirmed from a large prospective international cohort study (49, 50). Aardoom et al. further reported on average anemia and high platelet counts in the patients with IBD investigated. In IBD, ID and IDA caused by chronic bleeding, reduced intake or absorption of iron are common extra-intestinal complications (51). In adults, anemia was shown to be an independent risk factor for postoperative thrombosis in this high-risk patient group. Following the concept of multifactorial development of thrombosis (52), ID or IDA add to established risk factors as inflammation, steroid therapy, protein loss or dehydration (53, 54) further increasing thrombosis risk in children with IBD. These data support trials investigating the impact iron replacement in children with IBD has on the incidence of thromboembolic events (4, 55).

In children with cancer or renal disease, anemia in general does not result from ID e.g., in renal disease data suggest that anemia and correction with erythropoiesis stimulating agents are associated with increased risks for thromboembolic events (56). Thus, optimal treatment strategies to protect from thromboembolic events and the role prevention or treatment of ID and IDA need to be evaluated in clinical trials for specific patient groups.

## Prothrombotic risks in other forms of anemia

The association of anemia and thrombosis is more pronounced and has been well-described in other forms of congenital or acquired anemia. Pathogenesis of thrombosis in these diseases is complex and differs depending on the underlying disease. The impact of the common to all low hemoglobin values has not been well-investigated and may be of minor relevance. Growing evidence shows that chronic hemolytic anemia, hemoglobinopathies, and specifically sickle cell anemia (SCD) are primarily thromboinflammatory disorders as reflected by alterations both of inflammatory and coagulation pathways (57). Endothelial inflammation enhances leukocyte/ platelet adhesion by surface expression of adhesion molecules and release of prothrombotic von Willebrand factor and factor VIII. The role of hemolysis itself has come more into focus to contribute to procoagulant responses (58). The exposure of phosphatidylserine on the red blood cell (RBC) surface, the release of free hemoglobin and heme from damaged RBCs, and the shedding of RBC-derived microvesicles are well-established procoagulant factors (59). Moreover, splenectomy, which is performed in patients with specific diseases of chronic anemia as part of the therapeutic regimen, may further increase risks for arterial and venous thrombosis in chronic hemolytic anemia. Those risks may be attributed to loss of splenic filtering, increased platelet counts or free microparticles and are particularly high in thalassemia patients (60). Moreover, children with sickle cell disease or hemolytic anemia and severe anemia may developed stroke in the absence of thrombosis, which may be due to inability to deliver enough oxygen to brain tissue to meet metabolic demands (61).

Nutrient deficiencies other than ID, have been described to be associated with thrombosis as well. Vitamin B12 and folic acid deficiency lead to an increase in homocysteine. Hyperhomocysteinemia has been identified as an independent risk factor for arterial and venous thrombosis in childhood (62). The impact anemia has on thrombus development has not sufficiently been investigated so far.

## Limitations of the current evidence

Anemia is a common phenotype of various underlying diseases that influence thrombosis risk by complex mechanisms. Thus, investigating the association of anemia and thrombus development from observational studies is prone to confounding. Children with IDA are likely to have an underlying pathology, be that inadequate diet, bleeding or gastrointestinal malabsorption, which may be unknown at the time of thrombosis or may not be taken into account. As a result this pathology may confound the association, distort risk estimates and consequently poses an inherent limitation to the current conclusions.

Moreover, when comparing the pediatric data presented here to evidence from adult patient collectives it is important to keep in mind the significant differences in thrombosis etiology between adults and children. Among others, vascular diseases, diabetes, malignancies or obesity are substantially influenced by age.

In thrombocytosis, the etiology of platelet elevation needs to be analyzed in detail, as does the magnitude. In particular, the effect of thrombocytosis in myeloproliferative disorders on both bleeding and thrombosis is not transferable to thrombocytosis in children with iron deficiency.

Even though the experimental data reported add important information on the pathophysiology, further research is needed to validate the causal relationship of ID/IDA and thrombosis proposed here and disentangle the impact each ID, anemia and thrombocytosis independently have on thrombotic tendency.

## Conclusion

Because of the high prevalence, the association of iron deficiency anemia with the rare and potentially severe phenotype of pediatric thrombosis is clinically relevant. Data from animal models complement the clinical evidence and support that both IDA and secondary thrombocytosis are independent risk factors for thrombus development and propagation. Thrombosis development and recurrence may be substantially reduced by consequent prevention from or treatment of anemia. However, because many clinicians do not recognize anemia as a risk factor for thrombosis, iron replacement may be missed. In high-risk patient groups for thrombosis and anemia it is important to clarify, whether and to what extent iron-supplementation can prevent thrombosis and for whom thromboprophylaxis is indicated.

## Author contributions

HK and SH reviewed the literature, wrote and edited the manuscript. HC wrote and edited the manuscript. All authors agreed to the final version of the manuscript.

## Conflict of interest

The authors declare that the research was conducted in the absence of any commercial or financial relationships that could be construed as a potential conflict of interest.

## Publisher's note

All claims expressed in this article are solely those of the authors and do not necessarily represent those of their affiliated organizations, or those of the publisher, the editors and the reviewers. Any product that may be evaluated in this article, or claim that may be made by its manufacturer, is not guaranteed or endorsed by the publisher.
